# Effect of coronavirus disease 2019 on diagnosis and treatment of hepatocellular carcinoma: a systematic review

**DOI:** 10.37349/etat.2023.00179

**Published:** 2023-10-26

**Authors:** Afrooz Mazidimoradi, Samane Sabet Birjandi, Hamid Salehiniya

**Affiliations:** Università Politecnica Marche, Italy; Nicola Normanno, Istituto Nazionale Tumori-IRCCS-Fondazione G. Pascale, Italy; ^1^Student Research Committee, Shiraz University of Medical Sciences, Shiraz 71348-14336, Iran; ^2^Department of Midwifery, Birjand Branch Islamic Azad University, Birjand 97178-11111, Iran; ^3^Social Determinants of Health Research Center, Birjand University of Medical Sciences, Birjand 97178-53577, Iran

**Keywords:** Diagnosis, treatment, liver cancer, hepatocellular carcinoma, mortality, coronavirus disease 2019, systematic review

## Abstract

**Aim:**

Changes in strategies in the coronavirus disease 2019 (COVID-19) crisis and the imposing of restrictions have isolated many vulnerable patients including those with hepatocellular carcinoma (HCC) from routine medical care. This study investigated how the COVID-19 pandemic is affecting the diagnosis and treatment of HCC.

**Methods:**

An extensive search was conducted in the PubMed, Scopus, and Web of Science databases by using the appropriate keywords: COVID-19, hepatocellular carcinoma, hepatocellular cancer, and MeSH. Studies in English related to the purpose of the study were included in the analysis, and review studies, case reports, letters to editors, comments, and reports were excluded. The quality of the studies was assessed by the “Adapted Newcastle-Ottawa Quality Assessment Scales” checklist. The Endnote X7 software has been used for managing items.

**Results:**

The final qualitative analysis consisted of 27 articles. During the COVID-19 crisis, HCC diagnosis decreased from 20% to 34.13% compared to pre-crisis. The impact of the COVID-19 pandemic on HCC treatment encompasses a wide range of aspects. Generally, delays in treatment for patients with HCC ranged from more than one month for 21.5% of patients in France, to two months for 26% of patients in Italy, up to 30% in Austria, and 66.7% in Asia-Pacific countries.

**Conclusions:**

According to the findings, developing and implementing appropriate diagnostic and therapeutic strategies and developing low-cost and high-precision screening programs among high-risk populations seem to be effective in reducing the impact of the COVID-19 pandemic on HCC management.

## Introduction

During the global outbreak of the coronavirus disease 2019 (COVID-19) pandemic, healthcare systems around the world have focused on change to overcome the consequences, complications, and mortalities associated with COVID-19 [1]. Changes in strategies and restrictions have resulted in many health centers discontinuing routine care and placed vulnerable patients, including those with cancer, at significant risk [1, 2]. Routine screening programs for cancers such as breast, colon, cervical, etc. were stopped or faced serious challenges; the personnel of the cancer centers were transferred to the centers to fight against corona; the services of diagnostic centers were stopped or suspended and the treatment of patients was delayed. It is predicted that these delays will lead to a crisis in the post-corona era and will affect the world community by increasing the diagnosis of cancers at higher stages and reducing the life expectancy of cancer patients [3–7]. Hepatocellular carcinoma (HCC) is also one of the cancers that its diagnosis and treatment faced serious challenges during the COVID-19 pandemic.

HCC with 905,677 new cases and 830,180 deaths is the sixth most common cancer and the third leading cause of cancer death worldwide in 2020 [8] which is largely the problem in less developed areas [8, 9]. HCC accounts for 10.5% and 5.7% of all cancer deaths in men and women, respectively. The highest incidence of HCC (29.6%) has occurred in East Asian countries and the highest death rate (10.5%) in North African countries [8]. HCC with an unfavorable prognosis is highly invasive; and even with newer surgical interventions and treatment approaches, patients with HCC still have poor survival rates [10–12], as the overall mortality-to-incidence ratio is equal to 0.95 [8]. As a result, delays in diagnosing and treating this cancer can have irreparable consequences for patients, as it has been demonstrated that delays in screening or surgical procedures and treatments can increase the diagnosis of HCC in later stages by about 25% [13]. Delaying all urgent activities in health care centers, including reduced referrals to liver clinics, inadequate care for patients with HCC, and delays in liver transplantation (LT) activities are the most important consequences [14]. Therefore, considering the impacts of the COVID-19 pandemic on the diagnosis and treatment of HCC and the importance of this issue for decision-making in healthcare systems, as well as the fact that no comprehensive study has been conducted in this field, the present study reviewed the impacts of the COVID-19 pandemic on the diagnosis and treatment of HCC.

## Materials and methods

### Search strategy

This systematic review was carried out through the Preferred Reporting Items for Systematic Reviews and Meta-Analyses (PRISMA) checklist. A comprehensive search was performed in PubMed/MEDLINE, Scopus, and Web of Science databases, by using COVID-19, COVID-19 pandemic, coronavirus disease 2019, SARS-CoV-2, COVID-19 virus infections, SARS coronavirus 2 infection, COVID 19, liver neoplasms, liver cancer, and hepatocellular cancer keywords. AND, OR, and MeSH terms operators were also used to enhance the search result.

### Primary and secondary outcomes

The main outcome of this study was the impact of the COVID-19 pandemic on the diagnosis and treatment of HCC. The second result was the consequences of delay in diagnosing and treating HCC due to the COVID-19 pandemic.

### Inclusion and exclusion criteria

Included articles in this review, were all observational and interventional studies that investigated the impact of COVID-19 on HCC diagnosis and treatment that were published in the English language. Review studies, case reports, letters to editors, commentaries, and reports were excluded.

### Screening and selection of studies

All retrieved articles were entered in the Endnote X7 software after the search. In addition, duplicated articles were excluded by Endnote software. Then titles and abstracts were assessed, and those that were relevant to the aim of the review were included in the study. This phase was evaluated by two authors independently and articles with defined inclusion criteria were disagreements resolved by discussion between the two review authors, if no agreement could be reached, the third author gave them a consultation. Articles that examined the impact of COVID-19 disease on the diagnosis and treatment of HCC were eligible for analysis.

### Data synthesis and data extraction

We confined our analysis to descriptive measures of the consequences in each included review and presented the abstract of the result as the tables. The prepared checklist was used to extract the data and information such as the first author’s last name, the publication year, the country of study, the study type, and the sample size, as well as the major results presented by category in a separate table.

### Quality assessment

The quality of the articles was assessed by the “Adapted Newcastle–Ottawa Quality Assessment Scales” checklist [15]. This tool has three parts: selection, comparison, and conclusion. In addition, studies were divided into good, moderate, and poor categories, based on overall results. Two researchers independently assessed the reviews; if there wasn’t agreement, the third reviewer assessed it.

## Results

### Selection of studies

**Figure 1:**
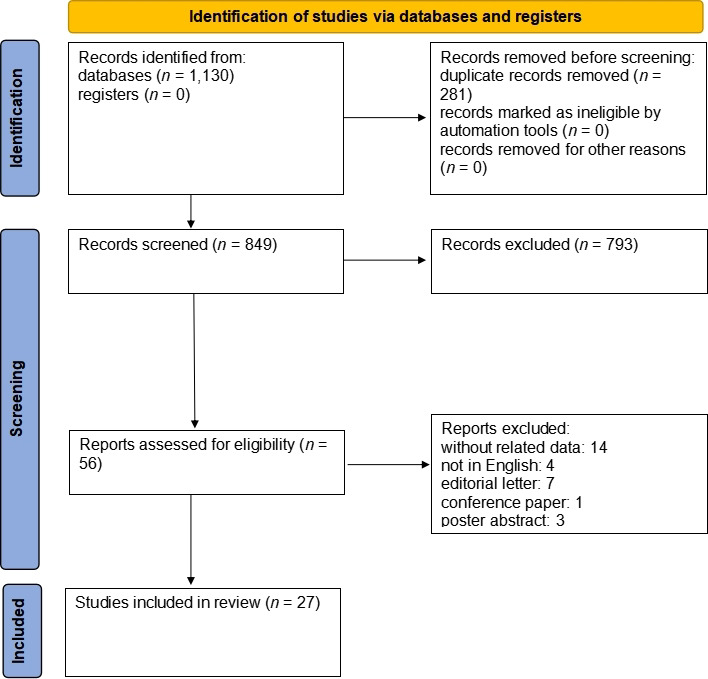
PRISMA flowchart of information through the systematic review phases

A total of 1,130 articles were retrieved as the search result. After removing duplicates (281 articles) and untied articles by title and abstract (793 articles), 58 articles remained. Then, by a review of the remaining articles; 29 other articles were excluded due to the following issues: data were not separated (12 articles), letters to the editor (7 articles), conference papers (1 article), poster abstract (3 articles), and non-English language articles (4 articles). After reviewing the article’s full text, 2 articles were removed because of lack of access to the full text, and finally, 27 articles remained for analysis in this study (Figure 1).

### Characteristics of included studies and quality assessment

Depending on the objective of the study, the included papers were divided into 2 main categories: the impact of the COVID-19 crisis on the diagnosis of HCC and the impact of the COVID-19 crisis on the treatment of HCC (Tables 1, 2, 3, and 4). The former was divided into 5 subgroups, including diagnosis of HCC in medical centres [16–22], activities of diagnostic centres and pathology laboratories [23–25], delay in diagnosis [18], screening method [26], and risk of HCC in patients with related alcohol-related hepatitis [27]. The latter was divided into 13 subgroups, including delaying or stopping treatment activities [18, 19, 22, 28–30], activities of medical centres [26, 28, 31–33], modifying treatment methods [18, 19, 29, 34], LT in patients with HCC [25, 29, 35–37], visiting and consulting patients [18, 19, 22, 25, 26, 29, 33, 38], hospitalization [29], performing treatment methods [19, 25, 29, 33, 37, 39, 40], pursue treatment by participating in research projects [19], therapeutic complications [18], response to treatment [41], follow up treatment [33, 42], the average time of performing treatment methods [25], and distribution of medicine [37]. Based on the result of the quality assessment, 17 articles had good quality, 4 had fair quality, and 4 had poor quality (Tables 1 and 2).

**Table 1:** The characteristics of articles included in a systematic review of the effect of COVID-19 on HCC diagnosis

**Reference**	**Place (country)**	**Sample size**	**Type of study**	**Review period or comparison date**	**Quality assessment**
Vigliar et al. [23]	Global (23 countries)	41 Cytopathology laboratories	International survey	The same period in 2020 *vs.* 2019 (different for each country)	Good
Amaddeo et al. [19]	France	6 Referral centers (670): 2020: 293 2019: 377	Retrospective and cross-sectional study	First 6 weeks of the COVID-19 pandemic (exposed) *vs.* the same period in 2019 (non-exposed)	Good
De Vincentiis et al. [16]	Italy	General hospital	Short report	11th Through the 20th week of 2018 to 2020	Good
Ferrara et al. [17]	Italy	7 Anatomic pathology units and secondary care hospital networks	NA	From weeks 11 to 20 of 2018, 2019, and 2020	Good
Gandhi et al. [18]	14 Asia-Pacific countries	27 Hospitals: 2,789 and 2,045 patients with new HCC diagnoses during the pre-and pandemic period	Survey	February to May 2019 *vs.* the same period in 2020	Good
Grinspan et al. [24]	New York	1,028 Pathology samples from 949 patients	Retrospective cohort study	February 1 to April 30, 2018 *vs.* same periods in 2019, 2020 and March 16, 2020 (as pre-COVID-19 and COVID-19 period in 2020)	Good
Iavarone et al. [25]	Italy	2019: 555 2020: 579	Retrospective study	2020 *vs.* 2019	Good
Kempf et al. [20]	France	First time: 450 in 2018, 398 in 2019, and 303 in 2020 Second time: 513 in 2018, 522 in 2019, and 469 in 2020	Prospectively	From January 1, 2018 to September 31, 2020	Good
Khan et al. [21]	US	41 HCOs	Retrospective multicenter research network study	From March 15, 2020 to July 15, 2020, and March 15, 2019 to July 15, 2019	Fair
Muñoz-Martínez et al [26]	76 Countries	76 Centres around the world	International survey	From May 2020 to June 2020	Fair
Perisetti et al. [27]	Global	23,201 Patients hospitalized with alcohol-related hepatitis	International survey	Between January 1, 2020, and December 1, 2020) *vs.* the same period in 2019	Good
Pomej et al. [22]	Austria	104 Males 22 Females	Retrospectively	Between December 1, 2019 and June 30, 2020	Good

HCOs: healthcare organizations; NA: not available

**Table 2:** The characteristics of articles included in a systematic review of the effect of COVID-19 on HCC treatment

**Reference**	**Place (country)**	**Sample size**	**Type of study**	**Review period or comparison date**	**Quality assessment**
Aghemo et al. [28]	Italy	194 Italian AISF members	Prospective web-based survey	From April 8, 2020 to May 3, 2020	Fair
Buonaguro et al. [34]	Naples and Italy	NA	NA	March 2020 compared to 2019	Poor
El Kassas et al. [42]	Egypt	NA	NA	Letter without data	Poor
Kounis et al. [35]	France	32 Patients with HCC	NA	Between April 15 and May 15, 2020 *vs.* the same time in 2019	Poor
Iavarone et al. [29]	Italy	2019: 117 2020: 77	NA	From February to March 20, 2020 *vs.* the same time in 2019	Poor
Mario et al. [39]	Italy	2019: 12 2020: 7	Single-center study	March 9 to April 30, 2019 *vs.* the same time in 2020	Fair
Martinez et al. [30]	Latin American	229 Centres	Global survey	From May 6 to May 30, 2020	Fair
Toyoda et al. [31]	US, Japan, and Singapore	3 Medical centers	Survey	March 1 to March 14, 2018, 2019, and 2020 and March 15 to May 1, 2018, 2019, and 2020	Fair
Amaddeo et al. [19]	France	6 Referral centers (670): 2020: 293 2019: 377	Retrospectiveand cross-sectional study	First 6 weeks of the COVID-19 pandemic (exposed) *vs.* the same period in 2019 (non-exposed)	Good
Bargellini et al. [40]	Italy	2019: 487 2020: 353	Retrospective	From the lockdown started until the phase of exiting the lockdown *vs.* the same time in 2019	Good
Crespo et al. [32]	Spain	81 Hospitals	Multicenter nationwide survey	Between March 30 and April 3, 2020	Good
Gandhi et al. [18]	14 Asia-Pacific countries	2,789 *vs.* 2,045 patients with new HCC diagnoses	Survey	February to May 2019 (pre-pandemic) *vs.* the same period in 2020 (pandemic period)	Good
Hartl et al. [38]	Austria	279 Cohort 1 and 138 cohort 2	Telesurvey	December 2019 to February 2020 and March to May 2020	Good
Jin et al. [41]	China	2020: 71, study group 2019: 83, control group	Retrospective	From January 2020 to March 2020 compared with the same period in 2019	Good
Iavarone et al. [25]	Italy	2019: 555 2020: 579	Retrospective study	2020 *vs.* 2019	Good
Muñoz-Martínez et al. [26]	76 Countries	76 Centers around the world	International survey	From May 2020 to June 2020	Fair
Pomej et al. [22]	Austria	104 Males 22 Females	Retrospectively	Between December 30, 2019 and June 30, 2020	Good
Ponziani et al. [37]	Italy	55 Different units present in 43 Italian hospitals	Online survey	January 15 to March 15, 2021 (second/third pandemic waves) *vs.* the same time in 2020	Good
Tan et al. [36]	Hong Kong (China) and Singapore	111	Modelling study	June 1, 2019 to May 30, 2020	Good
Zhao et al. [33]	China	42 Class-A tertiary hospital, 664 doctors	Nationwide online questionnaire survey	In COVID-19 period	Good

AISF: Association for the Study of the Liver

**Table 3:** The effect of COVID-19 on the diagnosis of HCC

**Category**	**Main findings**
Diagnosis of HCC in medical centers	HCC diagnoses in Italy fell in 2020 by 20% *vs.* 2018 and 2019 (from an average of 2.5 to 2 cases) [16] and fell in 2020 by 30.4% *vs.* 2018 and 2019 (from an average of 46 to 32 cases) [17] New HCC cases declined 26.7% during the pandemic *vs.* the pre-pandemic [18] The number of first diagnoses in patients affected by HCC declined (221 in 2020 *vs.* 304 in 2019) [19], and decreased over the weeks in 2020 but not in 2019 (*P* = 0.034), and the first diagnosis of HCC decreased (*P* = 0.083) [19] The number of new HCC cases declined 29% during the lockdown period in 2018, 2019, and 2020 (from 450 and 398 to 303) [20], and declined 9% after the lockdown period in 2018, 2019, and 2020 (from 513 and 522 to 469) [20] The average number of new diagnoses of liver and intrahepatic bile duct cancer declined 34.13% in the early phase of COVID-19 in 2020 *vs.* 2019 (from 13.8 cases to 9.09 cases per 100,000 patients with healthcare encounters) [21], and declined 25.58% in the late phase of COVID-19 in 2020 *vs.* 2019 (from 11.49 cases to 8.55 cases per 100,000 patients with healthcare encounters) [21] The number of new HCC diagnoses was equal two times (*n* = 14 *vs.* 14) [22]
Activities of diagnostic centers and pathology laboratories	The percentage of liver samples increased from 0.14% to 0.27% (*P* < 0.05) [23] Liver samples number decreased from 158 to 98 samples (*P* < 0.05) [23] The number of HCC diagnoses from February 1 to March 15, 2020 *vs.* 2018 and 2019 decreased from 80 and 27 to 15 cases [24], and from March 16 to April 30, 2020 *vs.* 2018 and 2019 decreased from 15 and 20 to 14 cases [24] The percentage of HCC diagnoses on February 1 to March 15, 2020 *vs.* 2018 and 2019 changed from 5.4% and 17.5% to 7.7% [24], and from March 16 to April 30, 2020 *vs.* 2018 and 2019 increased from 9.9% to 16.7% [24] In pre-COVID-19 2020 *vs.* during COVID-19 in 2020, the number of HCC diagnoses decreased from 15 cases to 14 cases [24], and % of HCC diagnoses increased from 7.7% to 16.7% [24] Cases discussed in MDTM 4.3% increase in 2020 *vs.* 82/555 (15%) in 2019 [25] HCC new diagnosis: 69/579 (12%) in 2020 *vs.* 82/555 (15%) in 2019 [25]
Delay in diagnosis	In BCLC 0/A/B recorded 48.2% and in BCLC C reported 51.9% [18]
Screening method	The screening program was changed in 80.9% of centers in the first wave of the COVID-19 pandemic [26] Biopsy and imaging technology requests were modified by 4.8% during the COVID-19 pandemic [26] The MR/CT acquisition strategy for HCC staging or assessment of response to treatment has been altered by 39.5% [26]
Risk of HCC in patients with alcohol-related hepatitis	HCC occurrence increased in the post-COVID group: OR = 1.19; 95% CI, 1.08–1.32; *P* < 0.001 [27]

MDTM: multidisciplinary team meeting; BCLC: Barcelona Clinic Liver Cancer; MR/CT: magnetic resonance/computed tomography; OR: odds ratio; CI: confidence interval

**Table 4:** The effect of the COVID-19 crisis on the treatment of HCC

**Category**	**Main findings**
Delaying or stopping treatment activities	Loco-regional surgical and nonsurgical treatment procedures reduced by 44% and 34%, respectively or suspended by 44% and 8%, respectively [28] Declined in patients starting treatment: reported by 27% of respondents [28] Systemic therapies were stopped by 4% and 34% did not have any major changes in their activity [28] Delay in the plan of HCC treatments for 2 months: 11 (26%) patients (2 TA, 4 TACE, 3 TARE, and 2 systemic therapies) [29] Treatment delay: 5 patients [30] More patients had a more than 1-month treatment delay in 2020 (21.5%) *vs.* 2019 (9.5%), *P* < 0.001 [19] More than 1 month delay rate in patients requiring an interventional procedure *vs.* those requiring medical treatment was higher (54.3% < 1 month, and 68.8% > 1 month *vs.* 41.8% < 1 month, and 13.8% > 1 month, respectively) [19] Treatment delays: 66.7% in BCLC 0/A/B and 63.0% in BCLC C [18] Higher visit delays in period 2: *n* = 31 (30%) *vs. n* = 10 (10%); *P* = 0.001 [22] Higher imaging delays in period 2: *n* = 25 (25%) *vs. n* = 7 (7%); *P* = 0.001 [22]
Activities of medical centers	In 45% of the centers, surveillance and follow-up were limited [28] Systemic therapies were stopped by 4% and 34% did not have any major changes in their activity [28] No significant decrease in the number of visits for patients with more advanced diseases (*P* trend = 0.11) [31] Between February 1 to March 14, 2020 and March 15 to May 1, 2020, the total number of HCC/cirrhosis visits 39.07% decreased (from 883 to 538); overall, 46.62% decreased (from 665 to 355) for the US site and 120 (26.6% decrease) for the Japan site [31] Gastroenterology and hepatology beds (40.7%), gastroenterologists (24.8%), and residents (58.3%) were allocated to COVID-19 patient care [32] Outpatient visits, abdominal ultrasounds, and endoscopies were reduced by 81.8–91.9% [32] Nine large university hospitals had 75% and 89% reductions in therapeutic endoscopies and HCC surgery, respectively, with the cancellation of elective liver transplants and transjugular intrahepatic portosystemic shunt [32] Imaging follow-up in HCC patients after treatment was changed by 73.5% of centers [26] Surgical treatments were rescheduled by 63.2% of centers [26] The ability to perform HCC treatments was maintained by 96% of centers [26] LT activity was not modified by 58.3% (28/48) of centers [26], 60.8% of centers (*n* = 45/76) were able to perform—surgical resections, 68.9% (*n* = 51/76) percutaneous treatments, and 81.1% (*n* = 60/76) loco-regional treatments [26] The option to initiate systemic treatment was maintained in 93.2% of the centers [26] In 50% of the centers (*n* = 38/76), curative and/or palliative treatments for HCC were canceled at least in 1 patient for each center because of SARS-CoV-2 infection [26] In 19 of 76 centers (51.4%), phone call visit service was modified: an increase in the number of calls (more days and/or more hours/day) was the most frequent modification in 84% of the centers, whereas 7 centers (17.9%) introduced phone call visits as a new practice during the COVID-19 pandemic [26] In the 58 centers that had nurses integrated into the HCC team, the liver-oncology nurses made decisions regarding face-to-face *vs.* phone call visits in 30.1% of the centers and organized the visits in 70.3% [26] The nurses undertook phone call visits 62.5%, to answer questions about treatment or follow-up events [26] Clinicians [51.4% (341/664)] and surgeons [57.6% (166/288)] reported more than a 60% reduction in the regular workload [33] During the pandemic, the regular workload reduced in 99.2% (659/664) of hospitals to varying degrees [33]
Modifying treatment methods	The surgical approach, with both minor and major resections, has been guaranteed in patients with liver metastases already treated with chemotherapy [34] Local ablation for primary and metastatic tumors has been performed regularly [34] TAs were carried out instead of preplanned surgical resection in three patients [29] Treatment strategy was modified in 13.1% of patients, with no differences between the 2 periods [19] The rate of treatments (proposed or performed) in patients with active HCC during the inclusion period was 56.7% (*n* = 377) in 2019 *vs.* 43.7% (*n* = 293) in 2020, with a significant decrease during the second half of the period in 2020 (*P* = 0.018) [19] A modification in the treatment strategy (between the treatments proposed during MTB and those finally received) was reported in 13.1% (*n* = 88) of patients, with no differences between the 2 periods [13.3% (*n* = 39) in 2020 *vs.* 13% (*n* = 49) in 2019; *P* = 0.91] [19] No differences in the treatment distribution: neither for the treatment intent (curative, palliative, or BSC) nor class (interventional, non-interventional, or BSC) [19] The main reasons for the modification of treatment strategy in 2020 *vs.* 2019: COVID-19 infection (46.1% in 2020 and 0% in 2019) and tumor progression (23.1% in 2020 and 65.3% in 2019) [19] Changes in treatment modality: 33.3% in BCLC 0/A/B and 18.5% in BCLC C [18]
LT in patients with liver cancer	Two (2.2%) patients dropped out of the waiting list [35] Liver transplants for HCC reduced from 3 in 2019 to 1 in 2020 [29] For nationwide LT waitlists in Hong Kong (China) and Singapore HCC dropouts at 1 year increased substantially by 31.8%, 107.96%, 176.06%, and 291.00% for a 1-, 3-, 6-, and 12-month disruption respectively [36] HCC LTs decreased by 35.7% (18 in 2020 *vs.* 28 in 2019) [25] Pre-LT evaluations were maintained in 41/55 (74.5%) of cases [37] LT activity was reduced by 44.4% (16/36) of centers [37] Post-LT follow-up reviews were unaffected in 27/38 (71.1%) of the centers [37] and urgent reviews were performed on 10/38 (26.3%) [37]
Visiting and consulting patients	Outpatient visits reduced from 117 in 2019 to 77 in 2020 [29] In 19 of 76 centers (51.4%), phone call visit service was modified: an increase in the number of calls (more days and/or more hours/day) was the most frequent modification in 84% of the centers, whereas 7 centers (17.9%) introduced phone call visits as a new practice during the COVID-19 pandemic [26] In the 58 centers which had nurses integrated into the HCC team, the liver-oncology nurses made decisions regarding face-to-face *vs.* phone call visits in 30.1% of the centers and organized the visits in 70.3% [26] The nurses undertook phone call visits in 62.5%, to answer questions about treatment or follow-up events [26] Outpatient visits decreased by 8.9% (1,416 in 2020 *vs.* 1,555 in 2019) [25] Cases discussed in multidisciplinary meetings reduced from 46 in 2019 to 42 in 2020 [29] A higher rate of consultations canceled, the outpatient models have changed with significantly greater use of teleconsultation during the pandemic [7.8% (*n* = 21) *vs.* 1.4% (*n* = 5), *P* < 0.001] [19] The percentage of remote consultations increased during the pandemic [35.9% (*n* = 105) *vs.* 1.3% (*n* = 5), *P* < 0.001, respectively] [19] The decline of 27.3% in face-to-face patient consultations [18] The increase of 18.3% in video/telephonic consultations [18] HCC patients [56.1% (*n* = 23/41)] reported significantly less telemedical contact with their hepatology specialist; *P* < 0.001 [18] HCC patients [75.6% (*n* = 31/41)] have fewer personal visits to the hospital [38] In patients diagnosed with HCC, acute medical help was required by 9/22 (40.9%) during healthcare restrictions related to COVID-19; *P* = 0.253 [38] Compared to the situation before COVID-19, 18.5% (4/22) HCC patients reported increased problems in searching for medical help [38] Face-to-face contact with the treating physician was low among HCC patients: VAS = 8.7 ± 1.7; *P* = 0.066) [38] Patient satisfaction with treatment of liver disease during COVID-19-related health care restrictions was minimal in patients with HCC (*n* = 40: –0.2 ± 0.9; *P* = 0.159) [38] Elective HCC admissions increased by +19.6% (*P* = 0.002) [38] Personal visits were reduced, and teleconsultation was increased [22] A median number of elective/non-elective admissions was not different between the periods [22] The hospitals [82.5% (548/664)] launched a remote consultation service for HCC patients during the COVID-19 outbreak, and most respondents [92.5% (614/664)] used online medical consultation to substitute for the “face-to-face” visits [33]
Hospitalization	The total number of patients admitted to the Ward reduced from 58 in 2019 to 48 in 2020 [29]
Performing treatment methods	Surgical resections reduced from 3 in 2019 to 2 in 2020 [29] The number of surgical procedures for HCC decreased from 12 to 7 [39] The percentage of surgical procedures for HCC increased from 14.2% to 18.9% [39] The rate of treatments (proposed or performed) in patients with active HCC during the inclusion period was 56.7% (*n* =377) in 2019 *vs.* 43.7% (*n* = 293) in 2020, with a significant decrease during the second half of the period in 2020 (*P* =0.018) [19] Decrease (20.2%) in TACE/TARE procedures: 146 in 2020 *vs.* 183 in 2019 [25] Surgical or locoregional treatments for HCC were reduced or stopped in a significant number of centers [29/52 (55.8%) and 25/52 (48.1%), respectively], with similar rates compared to the first wave [37] Systemic therapies were still prescribed by 36/49 (75.5%) of the centers [37] Reduction (27.5%) in the number of patients referred to MLTB (from 484 procedures in 2019 to 353 procedures in 2020) [40] Percutaneous ablations fell by 28.3% (from 60 procedures in 2019 to 43 procedures in 2020) [40] TACE was stable (63 procedures in 2019 and 64 in 2020) [40] SIRT increased by 64% (from 25 procedures in 22 patients in 2019 to 41 procedures in 36 patients in 2020) [40] In 2020, there were 31 (75.6%) primary lesions that were treated (mostly HCC), compared to 14 (56%) procedures in 2019 [40] Over 50% of HCC patients were in the intermediate stage (BCLC B), while approximately one-third of cases were in the advanced stage (BCLC C) due to intrahepatic macrovascular invasion [40] No early-stage HCC patients underwent SIRT between March and July 2020, compared to three (25%) cases treated in 2019 (*P* = 0.04) [40] Treatment modalities did not differ significantly comparing 2019 and 2020 [40] In 2020, the number of procedures performed using holmium-166-labeled microspheres increased (19.5% in 2020 compared to 9.1% in 2019) [40] A considerable amount of experts recommended non-surgical treatment strategies, including RFA (33.4%) and observation (23.6%) [33]
Pursue treatment by participating in research projects	A study protocol was accepted by 36 (5.4%) patients, with no differences in the inclusion rates between the 2 periods [4.1% (*n* = 12) *vs.* 6.4% (*n* = 24) in 2020 *vs.* 2019, respectively; *P* = 0.228] [19]
Therapeutic complications	Increase in treatment complications: about 15% across all BCLC stages [18]
Response to treatment	ORR after the latest radiologic treatment: 23.9% in the study group *vs.* 39.8% in the control group (*P* = 0.037) [41] Based on the ROC curve, the cut-off value to divide the follow-up interval into long- and short intervals is 95 days [41] Independent predictors for the efficacy of TACE treatment: grouping (OR = 2.402; 95% CI, 1.040-5.546; *P* = 0.040), long interval (OR = 2.573; 95% CI, 1.022–6.478; *P* = 0.045) and China HCC staging system (OR = 2.500; 95% CI, 1.797–3.480; *P* < 0.001) [41]
Follow up treatment	The median follow-up interval: 82.0 days (IQR, 61–109) in the study group *vs.* 66 days (IQR, 51–94) in the control group (*P* = 0.004) [41] For HCC patients who underwent routine postoperative follow-up after liver resection, 62.2% (178/286) of the surgeons recommended a follow-up when it is more than three months from the last review, while 15.4% (44/286) suggested a postponement in any case [33] For patients who received TACE, 55.4% (46/83) of the interventional oncologists recommended a follow-up delay of up to six months from the previous follow-up, while 20.5% (17/83) did not suggest a follow-up during the pandemic [33] Suggestion postponement or cancellation of the follow-up for patients who finished their radiotherapies by 86.1% (112/130) of clinicians [33]
The average time of performing treatment methods	Timeframes MDTM-TACE: 15 (2–112) days in 2020 *vs.* 20 (4–69) days in 2019; *P* = 0.42 [25] Timeframes for HCC treatment-radiological evaluation of response: 41 (16–162) days in 2020 *vs.* 34 (4–77) days in 2019; *P* < 0.0001 [25] Timeframes for outpatients’ visit-radiological evaluation of response: 69 (20–198) days in 2020 *vs.* 64 (26–161) days in 2019; *P* = 0.0006 [25]
Distribution of medicine	Home drug delivery was implemented by 14.5% of the centers [37]

TA: thermal ablation; TACE: transarterial chemoembolization; TARE: transarterial radio embolization; MLTB: multidisciplinary liver tumor board; SIRT: selective internal radiation therapy; RFA: radiofrequency ablation; BSC: best supportive care; MTB: multidisciplinary tumor board; VAS: visual analogue scale; ORR: overall response rate; ROC: receiver operator characteristic; IQR: interquartile range; SARS-CoV-2: severe acute respiratory syndrome coronavirus 2

### Effect of COVID-19 on the diagnosis of HCC

Based on the results of the studies, the impact of COVID-19 on the diagnosis of HCC can be summarized as follows.

#### Diagnosis of HCC in medical centers

In Italy, the average number of diagnoses of HCC in public hospitals decreased in 2020 compared with 2018 and 2019 by 20% [16], and another study showed it HCC decreased by 30.4% [17]. A study of HCC which was conducted in 27 hospitals in 14 countries in Oceania and Asia found that during the COVID-19 crisis, the diagnosis of new cases of HCC decreased by 26.7% [18].

In France, the number of patients with HCC, including patients with the first diagnosis was lower in 2020 (*n* = 221) than in 2019 (*n* = 304). Although this number decreased significantly during the weeks in 2020 with a similar trend to 2019 (*P* = 0.034), it was not significant for those who had their first diagnosis of HCC (*P* = 0.083) [19]. In addition, Kempf et al. [20] have shown that the diagnosis of HCC during the period of restrictions in 2020 compared with the same period in 2018 and 2019 decreased by 29%; and, after the removal of these restrictions, this downward trend also has continued.

In the US, the number of diagnoses of liver and intrahepatic parts of the biliary tract (C24) per 100,000 patients with healthcare encounters in the early phase of COVID-19 in 2020 decreased by 34.13% and in the delayed phase continued with a 25.58% decline [21]. In Austria, the number of cases of HCC diagnosed had no difference between 2019 and 2020 [22].

#### Activities of diagnostic centers and pathology laboratories

Global investigation showed that the number of pathology samples for HCC diagnosis decreased from 158 in 2019 to 98 in 2020, but the percentage of the pathology samples of HCC increased from 0.14% in 2019 to 0.27% in 2020 compared to the total pathology samples [23]. Grinspan et al. [24] found that the number of diagnosed HCCs decreased in 2020 compared to 2018 and 2019, but HCC compared to other gastrointestinal cancers, was higher in 2020. Also, during the COVID-19 period, the detection rate of HCC among gastrointestinal cancers increased from 7.7% to 16.7% [24]. Some investigations in Italy demonstrated that despite a 4.3% increase in the number of referrals to the MDTM, the diagnosis of new cases of HCC has decreased from 15% in 2019 to 12% in 2020 [25].

#### Delay in diagnosis

In 14 countries of Oceania and Asia, the diagnosis of HCC was delayed in 48.2% of BCLC cases 0/A/B and 59.1% of BCLC C cases [18].

#### Screening method

During the COVID-19 pandemic, 80.9% of 76 HCC diagnostic and treatment centers around the world HCC changed their screening programs during the first wave of the corona. Furthermore, 40.8% of centers changed biopsy and imaging diagnostic procedure requests and their timing during the COVID-19 pandemic. Also, 39.5% of the centers modified magnetic resonance/computed tomography scan strategy for HCC staging or evaluation of treatment response [26].

#### Risk of HCC in patients with alcohol-related hepatitis

Patients admitted in the post-crisis period of COVID-19 had a higher risk for elevating incidence of HCC (OR = 1.19; 95% CI: 1.08–1.32; *P* < 0.001), which was attributed to delay or inconsistent standard HCC monitoring due to the prevalence of COVID-19 [27].

More details were presented in Tables 1 and 3.

### Effect of the COVID-19 crisis on the treatment of HCC

The impact of COVID-19 on the treatment of HCC based on the results of the studies, can be summarized as follows.

#### Delaying or stopping treatment activities

A total of 27% of members of the Italian Liver Study Association reported that both locoregional surgical and non-surgical treatments were reduced (44% and 34%, respectively) or suspended (44% and 8%, respectively). Also, the number of patients to started treatment fell and 4% stopped systematic treatment [28]. In Italy, planning for HCC treatment was delayed for 42 patients, of which only 26% had a delay of 2 months: 2 TA, 4 TACE, 3 TARE, and 2 systemic treatments [29]. Furthermore, Martinez et al. [30] found that in 2020 the treatment of 5 patients with HCC was delayed for one month.

In France, after comparing HCC data in the first 6 weeks of a pandemic in 2020 with a similar period in 2019, it was shown that 21.5% of patients in 2020 experienced a delay of more than 1 month in treatment, while this rate in 2019, was 9.5% (*P* < 0.001). In the COVID-19 pandemic, a higher rate of patients requiring an interventional procedure experienced a delay of more than one month compared to those requiring medical treatment [intervention method: < 1 month, 54.3% (*n* = 100) *vs.* more than 1 month, 68.8% (*n* = 75); medical treatment: < 1 month, 41.8% (*n* = 77) *vs.* > 1 month, 13.8% (*n* = 15)] [19]. A study investigated by Gandhi et al. [18] showed that during the COVID-19 crisis, in 27 hospitals in 14 countries in Oceania and Asia, HCC treatment of 66.7% of BCLC 0/A/B cases and 63% of BCLC C was delayed. In addition, Pomej et al. [22] found that compared to 2019, the number of patients with delay in visits was 30% (*n* = 31) *vs.* 10% (*n* = 10) cases (*P* = 0.001); and the number of imaging delays was 25% (*n* = 25) *vs.* 7% (*n* = 7) cases (*P* = 0.001), which was higher in 2020.

#### Activities of medical centers

A total of 194 members of the Italian Liver Study Association reported that in 45% of centers, the care and follow-up of patients with HCC were limited. Also, 34% of them reported fundamental changes in their activities [28].

In three treatment centers in US, Singapore, and Japan, was found that there was generally no significant reduction in the number of visits for patients with HCC and/or cirrhosis (*P* trend = 0.11). Also, it was not observed in other parts of the world, although there was a significant downward trend at the US site (*P* trend = 0.094). However, between February 1 and March 14, 2020 and March 15 to May 1, 2020, the total number of HCC/cirrus visits decreased by 39.07% and 46.62% for the patients in the US and 26.6% for patients in Japan [31].

In Spain, 40.7% of gastrointestinal beds, 24.8% of gastroenterologists, and 58.3% of staff are dedicated to caring for patients with COVID-19, and outpatient visits; in addition, abdominal ultrasound and endoscopy decreased by 81.8–91.9%. Also, nine large university hospitals had 75% and 89% reductions in therapeutic endoscopies and surgery for HCC [32].

The results of an international study of 76 HCC diagnostic and treatment centers around the world during the COVID-19 crisis found that 73.5% of post-treatment centers changed imaging follow-up in HCC patients. A total of 63.2% of surgical treatment centers and 52.9% of locoregional treatment centers maintained their ability to perform HCC treatment in 96% of the centers. Of the 48 centers with LT programs before the COVID-19 pandemic, 58.3% did not change their LT activity. A total of 60.8% of the centers were able to perform surgical resection, 68.9% had percutaneous treatments, and 81.1% had locoregional. The option of initiating systemic treatment was maintained in 93.2% of the centers. In 50% of the centers, treatment and/or palliative care for HCC was canceled in at least one patient percenter due to SARS-CoV-2 infection [26].

In a study in China that analyzed the activity of 664 specialist physicians in 42 first-class teaching hospitals, 51.4% of physicians reported a reduction of more than 60% in regular workload, and surgeons (57.6%) announced the largest ratio of workload reduction (more than 60%). During the pandemic, regular workload decreased in 99.2% of the surveyed hospitals by varying degrees [33].

#### Modifying treatment method

In Nepal and Italy was shown that during the COVID-19 pandemic, surgical procedures were performed unchanged in patients with liver metastases. Local ablation for primary and metastatic tumors also has been performed regularly [34]. The results of a study in Italy revealed that in 3 patients TA was performed instead of pre-planned surgical resection [29].

In France, comparing HCC data in 2020 with 2019, it was shown that the treatment strategy changed in 13.1% of patients with no difference between the two periods [19]. Also, treatment strategy changes (between the proposed treatments during MTB and the treatments that were finally received) were reported in 13.1% of patients, with no difference between the two periods (*P* = 0.91). There were no differences in the distribution of treatment: neither for treatment (therapist, palliative, or BSC) nor for the group (interventional, non-interventional, or BSC). The main reasons for the alteration in treatment strategy in 2020 compared to 2019 were significantly different including COVID-19 infection, and tumor progression [19]. A study of HCC in 27 hospitals in 14 countries in Oceania and Asia demonstrated that in 33.3% of cases, BCLC 0/A /B and 18.5% of BCLC C treatment methods were changed [18].

#### LT in patients with HCC

In France, 2.2% of patients with HCC were excluded from the liver transplant waiting list during the COVID-19 crisis due to cancer progression [35]. Furthermore, in Italy, liver transplants for HCC dropped from three cases in 2019 to one case in 2020 [29]. A Chinese study of patients on the waiting list for liver transplants in Hong Kong (China) and Singapore predicted that by 2020, the number of cancellations of liver transplants in patients with HCC will increase significantly due to the disease progression of COVID-19 pandemic in one year (by 31.8%, 107.96%, 176.06%, and 291.00% for a 1-, 3-, 6- , and 12-disruptions respectively) [36]. In addition, HCC in Italy was shown that in 2020, compared to 2019, LT due to HCC decreased by 35.7% [25].

After the COVID-19 crisis in 55 wards in 43 Italian hospitals, preoperative assessments remained unchanged at 74.5%, and liver transplant activity reduced in 44.4% of centers. Follow-up, after LT was ineffective in 71.1% of the centers and immediate examinations, were performed in 26.3% [37].

#### Visiting and consulting patients

In Italy, the number of outpatient visits for HCC patients decreased from 117 in 2019 to 77 in 2020 [29]. The results of an international study HCC showed that during the COVID-19 crisis in 51.4% of centers, the telephone call service was improved, and an increase in the number of calls (more days and/or more hours per day) had the most alteration in 84% of centers, while 17.9% centers introduced telephone calls as a new method during COVID-19 pandemic [26]. In 58 centers, nurses were added to the HCC team, HCC nurses made decisions regarding face-to-face *vs.* phone call visits in 30.1% of the centers, and in 70.3% of cases, they organized visits. Nurses undertook telephone call visits in 62.5% to answer questions about treatment or follow-up events [26].

In another study in Italy, outpatient visits decreased by 8.9%, and out of a total of 1,416 visits to the HCC outpatient clinic, 7.2 % were done through video calling [25]. Another study in Italy revealed that face-to-face counseling for patients with HCC in multidisciplinary counseling sessions was reduced from 46 in 2019 to 42 in 2020 [29]. In France, more consultations were cancelled in 2020 compared to 2019 (7.8% *vs.* 1.4%, *P* < 0.001, respectively). The percentage of remote consultations increased during the pandemic (35.9% *vs.* 1.3%, *P* < 0.001, respectively) [19]. A study in Oceania and Asia found that face-to-face patient consultations decreased by 27.3% in hospitals, and telephone and video counseling increased by 18.3% [18].

In Austria, 56.1% of HCC patients reported less telemedical contact with their liver specialist (*P* < 0.001). Also, 75.6% had fewer personal visits to the hospital, and 40.9% needed acute medical care during COVID-19-related healthcare restrictions (*P* = 0.253). A total of 18.2% of patients reported an increase in difficulty seeking medical help compared to the situation before COVID-19. The VAS index for face-to-face contact with the treating physician was low among HCC patients (*P* = 0.066), and patient satisfaction with treatment of liver disease during COVID-19-related health care restrictions was minimal in patients with HCC (*P* = 0.159) [38]. Pomej et al. [22] found that although face-to-face visits decreased, there was an increase in telemedical counseling, and the median number of elective/non-elective admissions did not differ in different periods of 2020 and 2019. In addition, Zhao et al. [33] analyzed the activity of 664 specialist physicians in 42 first-class teaching hospitals, 82.5% of the hospitals in question provided remote counseling services to HCC patients during the COVID-19 outbreak and the majority of respondents (92.5%) used online medical counseling instead of face-to-face appointments.

#### Hospitalization

Iavarone et al. [29] found that the total number of HCC patients admitted to the ward decreased from 58 in 2019 to 48 in 2020.

#### Performing treatment methods

Iavarone et al. [29] demonstrated that liver resection due to cancer decreased from 3 cases in 2019 to 2 cases in 2020. According to another study in Italy, the number of surgeries performed to treat HCC decreased from 12 in 2019 to 7 in 2020, but the percentage of surgeries performed to treat HCC compared to all surgeries increased from 14.2% in 2019 to 18.9% in 2020 [39]. In addition, Amaddeo et al. [19] found that the number of treatments suggested or performed in active HCC patients referred to the center decreased from 56.7% in 2019 to 43.7% in 2020, which was significant in the second half of the period in 2020 (*P* = 0.018). In Italy, laparoscopic ablations and TACE/TARE procedures decreased by 52.6% and 20.2%, and surgical resection and percutaneous ablations increased by 43.8% and 108.3% respectively [25]. Examining the activity of 55 departments of 43 Italian hospitals the was shown that surgical or locoregional treatments for HCC, at similar rates to the first wave, were decreased or stopped in significant numbers of the centers (55.8% and 48.1%, respectively). A total of 75.5% of centers continued to prescribe systemic treatments [37].

Also, in Italy, a 27.5% and 28.3% decline was observed in the number of patients referring to MLTB and percutaneous ablations respectively, while TACE remained unchanged and SIRT increased by 64%. In 2020, 31 surgeries were performed on primary lesions (mostly HCC), compared to 14 surgeries in 2019. More than 50% of patients with HCC were in the intermediate stage (BCLC B); approximately one-third of cases were in the advanced stage (BCLC C) due to intrahepatic macrovascular invasion. No HCC patients underwent SIRT in the early stages between March and July 2020, while 3 cases were reported in 2019 (*P* = 0.04). The type of treatment in 2019 and 2020 was not significantly different; In 2020, the number of measures performed using the holmium-166-labeled microspheres method increased [40]. In China, oncologists were more concerned about antitumor effects, adverse drug reactions, quality of life, and the stability of treatment, prevention, and control of COVID-19 in patients with advanced HCC receiving systemic therapies. Only 34% of specialists recommended surgical resection during this period. In addition, a significant number of experts recommended non-surgical treatment strategies, including RFA and observation [33].

#### Pursue treatment by participating in research projects

In France in 2020, 5.4% (36/670) of patients agreed to be enrolled in the study protocol, which had no difference from the pre-COVID-19 period (*P* = 0.228) [19].

#### Therapeutic complications

Twenty-seven hospitals in 14 countries in Oceania and Asia found that in the COVID-19 crisis, treatment complications increased by 15% at all stages of BCLC [18].

#### Response to treatment

A study in China found that the ORR to treatment, after the last radiological treatment in the study group and the controls were 23.9% and 39.8%, respectively (*P* = 0.037) [41].

#### Follow-up treatment

A study in China showed that the mean follow-up interval (82 days) during the COVID-19 period was significantly higher than in the pre-pandemic period (66 days, *P* = 0.004) [41]. Among a total of 664 specialist physicians in 42 first-class teaching hospitals in China, 62.2% of surgeons, more than three months after the last examination recommended follow-up, for HCC patients who underwent routine postoperative follow-up after liver resection while 15.4% suggested postponement in either case. For patients receiving TACE, 55.4% of interventional oncologists recommend that patients can appropriately delay follow-up, but not more than six months from the previous follow-up, while 20.5% did not suggest follow-up during the pandemic. Most physicians (86.1%) recommended postponing or canceling follow-ups for patients who had completed their radiation therapy (patients did not receive adjuvant radiation therapy) [33].

#### Average time of performing treatment methods

In Italy, the average time for MDTM-TACE was reduced from 20 days in 2019 to 15 days in 2020 (*P* = 0.42), HCC treatment-radiological evaluation of response increased from 34 days in 2019 to 41 days in 2020 (*P* < 0.0001), and for outpatients’ visit—radiological evaluation of response increased from 64 days in 2019 to 69 days in 2020 [25].

#### Distribution of medicine

One year after the COVID-19 crisis, home delivery was performed by 14.5% of 55 wards in 43 Italian hospitals [37].

Please refer to Tables 2 and 4 for more details.

## Discussion

In the current study, the effect of the COVID-19 pandemic on the diagnosis and treatment of HCC was investigated by a systematic review, and the final analysis was performed based on the results of 28 articles that were related to the purpose of the study. Eight articles reviewed the effect of COVID-19 on the diagnosis of HCC, sixteen articles examined the effect of COVID-19 on the treatment of HCC, merely, and four articles examined the effect of COVID-19 on both the diagnosis and treatment of HCC.

Even though HCC is a major health problem in developing countries [8], the majority of the studies have been conducted in European countries (50%). Large-scale screening for HCC is not common. The prevalence of HCC is closely related to the prevalence of its risk factors such as hepatitis B and C and alcohol consumption [8]; so, extensive screening programs are performed only in countries with a high prevalence of risk factors among high-risk groups [43, 44]. There is a lack of studies evaluating the impact of the COVID-19 pandemic on large-scale liver cirrhosis (LC) screening in high-risk groups, and delay in diagnosis was evaluated by investigating the number of referrals, the amount of diagnosis during care, pathology specimens, or ultrasound procedures. In general, 80.9% of centers changed their screening programs during the first corona wave, and 40.8% of centers changed biopsy and imaging diagnostic procedures requests, and their timing during the COVID-19 epidemic [26].

The reduction in the diagnosis of HCC during the COVID-19 crisis has been reported in various studies from 20% [16] to 34.13% [21], and after the removal of restrictions, this downward trend has continued. Only in Austria, the diagnosis has been reported unchanged [22]. The results of three studies showed that despite the decrease in the number of pathology specimens, the diagnosis of HCC has a larger share among other cancers, which may be due to delays or reduced optimal care for at-risk patients during the COVID-19 crisis [23, 24]. Since HCC is a slow-growing tumor that takes 4 to 5 months for the tumor to progress doubled [45], the results of an international study showed that in patients with alcohol-related hepatitis hospitalization admitted during the post-crisis period of COVID-19, the risk of HCC increased by 19% [27]; these results emphasize the need to implement an HCC screening program in high-risk groups. In addition, it is predicted that the clinical impact of the missed diagnosis will be higher in long-term follow-up in the post-epidemic phase, and these results probably indicate only the tip of the iceberg [27]. Therefore, larger studies involving the hospitalized and outpatient populations may more appropriately address this issue and also recommend that care and treatment centers should be cautious for patients at risk of HCC to prevent missed ultrasound monitoring patients [27]. The findings of a study in Oceania and Asia countries revealed that the delay in diagnosis of HCC [18] is too high, and immediate interventions are essential to reduce this proportion.

The effect of the COVID-19 pandemic on the treatment of HCC encompasses a wide range of aspects; the method of visiting and consulting, activities of medical centers and doctors, delaying and stopping the implementation of treatment or changing its method, hospitalization, treatment duration, treatment through LT, treatment complications, patient follow-up and their access to the required medications.

Delays in starting treatment for HCC patients were reported for more than one month for 21.5% of patients in France [19], two months for 26% of patients in Italy [29], up to 30% in Austria [22], and 66.7% in 14 Asia-Pacific countries [18]. This is even though before the COVID-19 crisis, unlike cancers such as breast, lung, and prostate, the death rate of HCC has been increasing [46]. Increased exposure to risk factors acquired immunodeficiency syndrome (AIDS), hepatitis B, hepatitis C, alcohol consumption, etc. [47], lack of general, accessible, and inexpensive screening method [48, 49], diagnosis at higher stages, delay in receiving services treatment, older age, clinically significant portal hypertension (CSPH), early recurrence, and late recurrence are the main reasons for the increase in HCC mortality [50]. With the emergence of the COVID-19 crisis, in addition to the order and warning to stay at home and the fear of contracting COVID-19 [6, 51], the access people to perform diagnostic and treatment services due to the effect of the COVID-19 pandemic on the activity of HCC treatment centers includes a reduction of workload of gastroenterologists [33], restriction and changes in activities [28], allocation of gastrointestinal beds, gastroenterologists, and staff to COVID-19 [32], reducing outpatient visits, abdominal ultrasound, endoscopy, and reducing or canceling various treatments [26, 32] faced a challenge. Therefore, it is expected that in the post-corona era, we will see a higher rate of death in patients with HCC.

Only three medical centers in the US, Singapore, and Japan did not show a significant reduction in visits of HCC patients [31]. The results of an international study showed that 96% of centers around the world have been able to maintain their ability to treat HCC [26]. These centers have considered the increase in remote medical visits and consultations (telephone or video calls) [18, 19, 22, 26] and the launch of remote consultation systems [33] as factors influencing the lack of change in their activities. But it should be kept in mind that these results are premature result and the late consequences of COVID-19 require more studies in the future because, in these same centers, patients were admitted late and imaging was stopped during lockdown periods [51], and also, patients have reported that it is difficult for them to access doctors for consultation even remotely [38]. Implementation of a home drug delivery system was another strategy for reducing the patient care lost [37].

Various studies showed that the decision-making for treatment methods for HCC patients during the COVID-19 pandemic is different from no change to replacement with less aggressive methods [18, 19, 29, 34]. Choosing the appropriate treatment method or a combination of treatments depends on the stage of the tumor, the function of the remaining liver parenchyma, the volume of the remaining liver in the future, and the general condition of the patient [52]. While orthotopic LT (OLT) and surgical resection are the only two curative options, OLT is the first line of treatment and the best treatment strategy because it not only removes the tumor but also treats the underlying liver disease [52]. As the application of OLT is currently limited by organ shortage, major liver resection—even in patients with underlying chronic liver disease—is increasingly accepted in clinical practice [53].

The prognosis of patients with HCC depends on the stage of the tumor, and only patients who are diagnosed in the early stages will benefit from treatment. LT or surgical resection of patients diagnosed at early stages can increase 5-year survival by 70%, while patients with advanced HCC are only eligible for palliative treatments and have a median survival of less than 1 year [54, 55]. Minimally invasive liver resection (MILS) has become an attractive option due to reduced intraoperative blood loss, shorter hospital stay, and similar oncologic outcomes compared to open liver resection. Nevertheless, the safety of MILS is still debated in challenging situations, such as cirrhotic patients, difficult tumor sites, multiple or large tumors, and re-resection [53].

Elimination of LT in patients with LC due to the disease progression in France has been reported at 2.2% [29]. Furthermore, decreases in liver transplants have been reported from 35.7% [25] to 44.4% [37] and it is predicted that in 2020 the number of transplant recipients in patients with HCC due to the progression of the disease will increase significantly due to COVID-19 pandemic [36].

All studies that have examined the status of surgical treatment have reported that the use of surgical techniques has decreased during the COVID-19 pandemic [25, 29, 37, 39]. The mean time of treatment methods except the MDTM-TACE method has increased [25]. The number of hospitalizations and the average follow-up interval between patients for treatment during the COVID-19 crisis showed that this has been done more with physicians’ advice to reduce the consequences of morbidity to COVID-19 [33, 41].

In general, the COVID-19 pandemic poses serious challenges in the diagnosis and treatment of HCC. However, given that by early diagnosis the chance of survival of patients with HCC doubles (OR = 2.08; 95% CI, 1.80–2.37), and with proper treatment, it becomes 2.24 times (OR = 2.24; 95% CI, 1.99–2.52) [56]. It seems that the necessary measures should be taken during the COVID-19 crisis to reduce patients’ fears of infection and increase timely receipt of services by them; the establishment of an appropriate triage system to prevent and control the transmission of infection through asymptomatic patients, including temperature measurement, screening of symptoms and suspected contact, and measurement of blood oxygen levels of clients and isolating people suspected of having an infection from other patients can be useful [57]. Also considering the appropriate planning to take compensatory measures during the reduction of the severity of the epidemic and after its cessation is necessary to reduce its consequences on the timely diagnosis and treatment of patients with HCC, especially in high-risk areas.

The results of the studies emphasize the existence of serious challenges in the diagnosis and treatment of HCC during the COVID-19 pandemic, especially in patients with risk factors and those who require LT. Therefore, diagnostic and care systems should attempt the design, development, and implementation of appropriate and rapid diagnostic with a focus on emerging imaging techniques and biomarkers for early disease diagnosis; and select therapeutic strategies based on the tumor stage, the function of the remaining liver parenchyma, the future liver remnant volume and the patient’s general condition. Also, the implementation of low-cost and high-precision screening programs among high-risk populations and communities during the period of the COVID-19 pandemic and when the severity of the epidemic is decreasing.

## Abbreviations

BCLC: Barcelona Clinic Liver Cancer
BSC: best supportive care
CI: confidence interval
COVID-19: coronavirus disease 2019
HCC: hepatocellular carcinoma
LT: liver transplantation
MDTM: multidisciplinary team meeting
OLT: orthotopic liver transplantation
OR: odds ratio
SARS-CoV-2: severe acute respiratory syndrome coronavirus 2
SIRT: selective internal radiation therapy
TA: thermal ablation
TACE: transarterial chemoembolization
TARE: transarterial radio embolization

## Declarations

### Copyright

© The Author(s) 2023.
